# Advancements in research on feeding intolerance to enteral nutrition in neurocritical patients undergoing sedation and analgesia

**DOI:** 10.3389/fnut.2026.1849390

**Published:** 2026-07-30

**Authors:** Na Lu, Zhao Qi Du, Xue Chang Cai, Shui Hua Jin, Yue Zhang

**Affiliations:** 1 Department of Clinical Nutrition, Qingdao Huangdao District Central Hospital, Qingdao, China; 2 Department of Neurosurgery, Qingdao Huangdao District Central Hospital, Qingdao, China

**Keywords:** dietary fiber, enteral nutrition, feeding intolerance, intra-abdominal pressure, neurocritical care, review, sedation and analgesia

## Abstract

In the Neurocritical Care Unit (NICU), deep sedation and analgesia are essential for managing critically ill patients to control intracranial pressure and reduce brain stress. However, these drugs—particularly opioids and benzodiazepines—significantly impair gastrointestinal motility and barrier function, increasing the risk of feeding intolerance (FI) to 46.38–62.0% in this population (1). FI manifests as elevated gastric residual volume, abdominal distension, vomiting, or failure to meet enteral nutrition targets within 72 h (2), and is associated with higher infection rates, prolonged hospital stay, increased medical costs, and worse neurological and overall prognosis (3). Therefore, clarifying the pathophysiological mechanisms, risk factors, assessment methods, and management strategies of FI in the context of sedation and analgesia is vital for developing standardized, individualized nutritional support plans for neurocritical patients. This review systematically elaborates the “neuro-sedation-gut” triad mechanism, stratifies population-specific risk factors graded by the GRADE system, integrates precise multimodal assessment tools, and proposes an algorithmic management pathway aligned with 2023–2024 ESPEN/ASPEN guidelines, to provide targeted theoretical and practical references for neurocritical care practice.

## 1 Introduction

Neurocritically ill patients with primary or secondary brain injury (including traumatic brain injury, intracranial hemorrhage, and severe stroke) commonly exist in a state of extreme systemic stress characterized by elevated intracranial pressure (ICP), excessive catecholamine release, and widespread inflammatory activation. Sedation and analgesia constitute foundational supportive therapy in the Neurocritical Care Unit (NICU) to reduce cerebral metabolic demand, suppress nociceptive stimulation, and prevent secondary brain injury. However, these interventions create a core clinical contradiction: μ-opioid receptor agonists and GABAergic agents (e.g., benzodiazepines, propofol) that protect the brain directly inhibit enteric nervous system activity, while the underlying cerebral pathology itself impairs splanchnic perfusion—jointly driving FI onset. FI is not an isolated gastrointestinal complication, but a key mediator of poor prognosis: intestinal barrier failure promotes bacterial translocation and amplifies systemic inflammation, which in turn exacerbates cerebral edema and neuronal damage, creating a vicious cycle that undermines the efficacy of neurosurgical and neuromedical treatment (1). Prior evidence confirms that unmanaged FI independently increases 28-day mortality in neurocritical patients by 37% (2). Therefore, FI management should be regarded as an integral component of holistic neurocritical care, rather than a peripheral nutritional technical issue. This review systematically synthesizes the latest advances in FI pathogenesis, risk stratification, monitoring, and intervention specific to sedated neurocritical populations, to bridge the gap between mechanistic evidence and bedside practice.

## 2 The definition and pathophysiological mechanism of FI

### 2.1 The definition of FI

Currently, the international consensus definition of FI in critically ill populations issued by the European Society for Intensive Care Medicine (ESICM) is widely adopted for neurocritical patients (3), and is defined by any of the following: ➀ Enteral nutrition is interrupted or reduced due to significant clinical gastrointestinal adverse reactions, such as vomiting, gastric residual volume (GRV) exceeding 250–500 mL, abdominal distension, or diarrhea; ➁ Despite active efforts, the daily energy intake target of 20 kcal/kg via the intestinal route cannot be achieved within 72 h of initiating enteral nutrition; ➂ Enteral feeding must be discontinued due to clinical complications, such as a strong suspicion of intestinal ischemia (4).

### 2.2 Pathophysiological mechanisms of FI under sedation and analgesia

The occurrence of FI in sedated neurocritical patients arises from the interaction of three core pathways.

#### 2.2.1 Neuro-humoral dysregulation driven by primary brain injury

**FIGURE 1:**
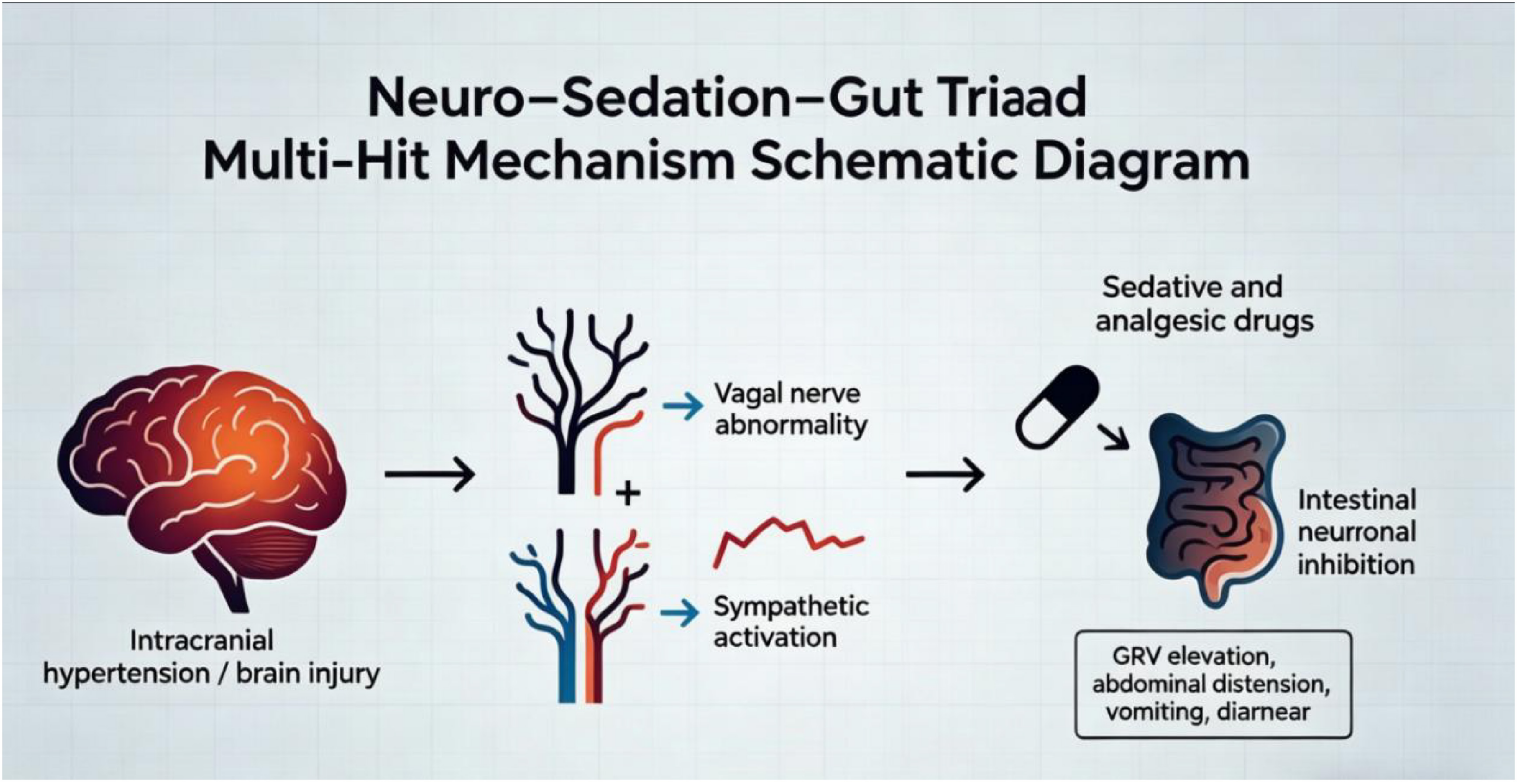
Multi-hit mechanism schematic diagram of neuro-sedation-gut triad.

The primary mechanism linking central nervous system injury to FI is autonomic nervous system imbalance triggered by intracranial hypertension: elevated ICP activates the sympathetic nervous system and suppresses vagal tone, leading to mesenteric vasoconstriction, intestinal hypoperfusion, and disruption of the intestinal mucosal barrier. This baseline injury is then exacerbated by sedative-analgesic agents: opioids activate μ-opioid receptors on enteric neurons, while benzodiazepines and propofol enhance GABAergic inhibition, jointly delaying gastric emptying, slowing intestinal transit, and reducing digestive secretion. The combined effect drives functional ileus, bacterial translocation, and progressive FI (5–10) (Figure 1).

#### 2.2.2 Dose-dependent effect of sedation depth

A clear dose-response relationship exists between sedation depth and FI risk: when the Richmond Agitation-Sedation Scale (RASS) score is ≤ -3, indicative of deep sedation, there is a dose-dependent increase in gastrointestinal motility inhibition. This results in a 2.1–3.5-fold higher risk of delayed gastric emptying compared to mild sedation (RASS −1 to −2). Furthermore, for each 1 μg/(kg⋅h) increment in opioid dosage, the incidence of FI increases by 15–20% (1–3, 11).

#### 2.2.3 Evidence update on sedation and feeding compatibility

The 2023–2024 ESPEN guidelines, supported by multiple randomized controlled trials (RCTs) conducted in neurocritical care populations, affirm that administering low-dose continuous enteral nutrition (20–30 mL/h) is both safe and feasible during daily sedation awakening trials (SAT), without elevating the risk of reflux or intracranial pressure (4, 12, 13).

## 3 Stratified risk factors and evidence levels for FI

Previous multivariable logistic regression analyses have consistently identified 11 independent predictors of FI within NICU populations (14–16). These factors are categorized into three domains that correspond to neurocritical care characteristics, and the evidence level is assessed using the GRADE system (Table 1).

**TABLE 1:** Stratified risk factors and evidence levels for FI.

Neurological factors in the patient	Risk factors	Impact on FI	Grade	Mechanism	References
	GCS ≤ 8 score	Independent risk factor OR = 2.31	A	Impaired consciousness → Weakened swallowing reflex and gastrointestinal regulatory dysfunction	(1, 6)
	ICP > 20 mmHg	Independent risk factor OR = 2.74	A	Intracranial hypertension → vagal suppression, intestinal hypoperfusion	(1, 6, 17, 35)
	Mild hypothermia therapy	Independent risk factor OR = 1.96	B	Low temperatures inhibit gastrointestinal peristalsis and metabolism, with gradual recovery after rewarming.	(1, 6, 17)
	APACHE II ≥ 20 score	Independent risk factor OR = 3.02	A	Severe stress → Inadequate systemic perfusion and impaired gastrointestinal function	(6, 18)
	Lactic acid in blood > 2 mmoL/L	Independent risk factor OR = 2.58	A	Low perfusion → intestinal ischemia and hypoxia, impaired absorption	(6, 18, 19)
	Hypoproteinemia (Alb < 30 g/L)	Independent risk factor OR = 2.17	A	Intestinal mucosal edema and impaired barrier function	(6, 18, 19)
	Elderly( ≥ 65 age)	Independent risk factor OR = 1.83	B	Physiological gastrointestinal hypofunction	(6, 18, 19)
Drug-related factors	Opioids (morphine/fentanyl)	Strong independent risk factor OR = 3.86A	A	Activation of μ receptors → inhibition of gastrointestinal peristalsis and delayed gastric emptying	(13, 19)
	Benzodiazepines	Independent risk factor OR = 2.25	A	GABAergic inhibition → dysfunction of the enteric nervous system	(6, 20)
	Propofol	Moderate risk factors OR = 1.79	B	Mildly inhibits gastrointestinal motility; less potent than benzodiazepines.	(4, 6)
	Dexmedetomidine	Protective Factor OR = 0.68	A	It exhibits the least gastrointestinal motility inhibition and is recommended as the first-line choice.	(1, 19)
Treatment-related factors	IAP ≥ 12 mmHg	Strong independent risk factor OR = 3.15	A	Compression of mesenteric vessels → decreased intestinal perfusion and weakened peristalsis	(17, 19)
	Mechanical ventilation	Independent risk factor OR = 2.43	A	Increased intrathoracic pressure → Impaired abdominal reflux, elevated IAP	(6, 19, 24)
	Vasoactive agent	Independent risk factor OR = 2.08	B	Constriction of visceral blood vessels → intestinal ischemia	(4, 6, 24)
	Supine feeding	Risk factors OR = 1.92	B	The risk of reflux aspiration increases, indirectly exacerbating FI.	(19, 20)

### 3.1 Neurological and disease-severity factors

#### 3.1.1 Intracranial hypertension (ICP > 20 mmHg)

Grade A evidence (1, 6, 17). Elevated ICP directly suppresses vagal activity and reduces splanchnic perfusion, representing a neurospecific risk distinct from general ICU populations.

#### 3.1.2 Reduced consciousness (GCS ≤ 8)

Grade A evidence (1, 6). Impaired neuroregulation weakens swallowing coordination and gastrointestinal autonomic control.

#### 3.1.3 Mild therapeutic hypothermia

Grade B evidence (1, 6, 17). Hypothermia directly slows smooth muscle contraction and delays gastrointestinal metabolic recovery, with function gradually restored after rewarming.

#### 3.1.4 High disease severity (APACHE II ≥ 20)

Grade A evidence (6, 18). Systemic stress response and multiorgan hypoperfusion preferentially impair the highly oxygen-sensitive gastrointestinal mucosa.

#### 3.1.5 Tissue hypoperfusion (blood lactate > 2 mmoL/L)

Grade A evidence (6, 18, 19). Persistent hyperlactatemia indicates occult intestinal ischemia, directly compromising absorption capacity.

### 3.2 Pharmacological factors

Opioids (morphine/fentanyl) have been identified as the most significant modifiable risk factor in sedated NICU patients, with Grade A evidence (13, 19). These drugs exert their effects through direct μ-receptor-mediated inhibition of enteric motility.

Benzodiazepines, supported by Grade A evidence (6, 20), enhance both central and peripheral GABAergic inhibition, leading to more pronounced gastrointestinal dysfunction compared to propofol.

Propofol, with Grade B evidence (4, 6), causes mild inhibition of motility and presents a lower risk than benzodiazepines. Dexmedetomidine is considered a Grade A protective factor (1, 19). As an α_2_-receptor agonist with minimal gastrointestinal impact, it is recommended as the first-line sedative for neurocritical patients at high risk for FI.

### 3.3 Treatment-related factors

#### 3.3.1 Intra-abdominal hypertension (IAP ≥ 12 mmHg)

Grade A evidence (17, 19). Compresses mesenteric vessels, worsens intestinal edema, and creates a feedback loop with impaired gastrointestinal motility. Routine bladder-pressure IAP monitoring in high-risk NICU patients is recommended by both WSACS and ESICM guidelines, and prospective cohort data show IAP elevation precedes overt FI symptoms by 12–24 h, supporting its role in early warning signs of FI (21–23).

#### 3.3.2 Mechanical ventilation

Grade A evidence (6, 19). Elevated intrathoracic pressure reduces mesenteric venous return and raises IAP.

#### 3.3.3 Vasoactive agents

Grade B evidence (4, 6, 24). Constrict splanchnic vasculature, exacerbating intestinal hypoperfusion.

#### 3.3.4 Supine positioning during feeding

Grade B evidence (19, 20). Increases reflux risk, indirectly aggravating gastrointestinal intolerance.

Additional general risk factors including hypoalbuminemia ( < 30 g/L) and age ≥ 65 years should be co-assessed in clinical practice (6, 18, 19).

## 4 The systematic framework for assessment and monitoring of FI

The development of a prospective and multi-faceted evaluation system constitutes the foundational element of FI management (17, 25, 26). Feeding intolerance monitoring around core risk domains to enable closed-loop management, combining conventional bedside metrics with novel quantitative tools:

### 4.1 Monitoring of intra-abdominal pressure (IAP)

As outlined in section 2.1, IAP is both a key risk factor and a core monitoring metric. Measurement follows the standardized bladder pressure method (21, 23): place the patient in supine position, empty the bladder, instill 25 mL sterile normal saline via the urinary catheter, align the zero reference point at the mid-axillary line of the iliac crest, and record the water column height at end-expiration (1 mmHg = 0.133 kPa). Where feasible, healthcare facilities may also employ direct monitoring via an external device attached to the pressure measurement tube. Research conducted by Bejarano et al. (27) has demonstrated a correlation between IAP and the tolerance of enteral nutrition in critically ill patients. Specifically, when IAP persistently exceeds 20 mmHg, the risk of feeding intolerance increases by a factor of 2.7, and there is a potential for the development of abdominal compartment syndrome (ACS) (28). It is advisable for patients at elevated risk of feeding intolerance or exhibiting signs of abdominal distension to undergo routine IAP monitoring at intervals of no less than every 4–6 h, based on the intra-abdominal pressure (IAP) levels. The feeding strategy should be adjusted accordingly. Management thresholds are:IAP 12–15 mmHg: Continue routine enteral nutrition;

IAP 16–20 mmHg: Switch to trophic feeding (10–20 mL/h); IAP > 20 mmHg: Hold enteral nutrition to prevent abdominal compartment syndrome (15, 21).

### 4.2 Monitoring of gastric residual volume (GRV)

Despite ongoing debate regarding the optimal threshold for gastric residual volume (GRV), typically ranging from 200 to 500 mL, dynamic monitoring of GRV trends remains a prevalent approach for assessing gastric emptying function. GRV can be assessed through the syringe aspiration method or via gastric ultrasound monitoring. The use of bedside ultrasound for GRV monitoring is increasingly favored over the traditional aspiration method due to its non-invasive nature, repeatability, and ability to avoid interruptions in feeding (26, 29, 30). Current recommendations suggest: GRV < 200 mL: Continue and advance enteral nutrition; GRV 200–500 mL: Maintain current rate or switch to trophic feeding; GRV > 500 mL: Hold enteral nutrition and re-evaluate (25, 31).

### 4.3 Standardized feeding tolerance assessment tool

A standardized scoring system, incorporating indicators such as abdominal distension, diarrhea, and gastric residual volume (GRV) (19, 32), including the Acute Gastrointestinal Injury (AGI) grading (33) (Table 2) and the enteral nutrition tolerance score (Table 3), is employed to quantitatively evaluate feeding intolerance (FI) (11, 34). The assessment of gastrointestinal function is categorized as follows: ➀ Normal gastrointestinal function: 0 points; ➁ Mild gastrointestinal dysfunction: 1–2 points; ➂ Moderate gastrointestinal dysfunction: 3–4 points; ➃ Severe gastrointestinal dysfunction: 5 points or more. In the context of enteral nutrition, when feeding intolerance is observed, the enteral nutrition tolerance score should be reassessed every 6–8 h. Adjustments to the enteral nutrition infusion should be made based on the scoring outcomes: ➀ If the score increase is ≤ 1 point, continue enteral nutrition and increase the infusion rate; ➁ If the score increase is 2–3 points, continue enteral nutrition, maintain the current infusion rate or reduce it, and provide symptomatic treatment; ➂ If the score increase is ≥ 4 points or the total score is ≥ 5 points, suspend enteral nutrition and implement appropriate interventions.

**TABLE 2:** Acute gastrointestinal injury (AGI) grading scale.

Grade	Definition	Clinical diagnosis or manifestations
No acute gastrointestinal injury	No gastrointestinal system dysfunction	No gastrointestinal symptoms
Acute GI injury Grade I: At risk of developing gastrointestinal dysfunction or failure	Partial impairment of gastrointestinal function, presenting with known-cause or transient gastrointestinal symptoms	Clinically evident gastrointestinal symptoms occurring after injury, which are transient and self-limiting. Examples: Nausea and vomiting on postoperative day 1 after abdominal surgery, absent bowel sounds postoperatively, shock, and early reduced bowel motility.
Acute GI injury, Grade II: gastrointestinal dysfunction	The digestive tract cannot fully perform digestion and absorption to meet the body’s nutrient and fluid requirements	Acute-onset gastrointestinal symptoms requiring clinical intervention to ensure adequate nutrition and fluid intake. Severe clinical status beyond expected levels in patients without prior gastrointestinal intervention or abdominal surgery. Examples: Gastroparesis with gastric retention or reflux, lower gastrointestinal paralysis, diarrhea, Grade I intra-abdominal hypertension (IAH) [intra-abdominal pressure (IAP): 12–15 mmHg (1 mmHg = 0.133 kPa)], visible blood in gastric aspirate or stool. Feeding intolerance is defined as enteral nutrition intake < 20 kcal/(kg/d) after > 72 h of feeding attempts.
Acute GI injury Grade III: gastrointestinal failure	Loss of gastrointestinal function; failure to recover gastrointestinal function despite clinical intervention, with no improvement in general condition	Diagnosis: Persistent enteral feeding intolerance despite clinical intervention (e.g., erythromycin administration, postpyloric tube placement), leading to persistent or worsening multiple organ dysfunction syndrome (MODS). Examples: Persistent feeding intolerance despite treatment (manifested as severe gastric retention), persistent gastrointestinal paralysis, new or progressive bowel dilatation, progression to Grade II IAH (IAP: 15–20 mmHg), and low abdominal perfusion pressure (APP < 60 mmHg). Feeding intolerance is present and may be associated with persistent or worsening MODS.
Acute GI injury Grade IV: gastrointestinal failure with severe impact on other organ function	Acute GI injury progresses to an immediately life-threatening severe state, accompanied by multiple organ dysfunction syndrome and aggravated shock	Diagnosis: Acute GI injury leading to severe, acute deterioration of systemic status, with multiple organ dysfunction and shock. Examples: Intestinal ischemia and necrosis, gastrointestinal bleeding leading to hemorrhagic signs and shock, Ogilvie’s syndrome (acute colonic pseudo-obstruction), abdominal compartment syndrome requiring decompression, etc.

**TABLE 3:** Enteral nutrition tolerance scoring scale for critically Ill neurosurgical patients.

Assessment item	0 Points	1 Point	2 Points	5 Points
Abdominal distension/abdominal pain	None	Mild abdominal distension without pain	Obvious abdominal distension or pain that resolves spontaneously, or intra-abdominal pressure (IAP) 15–20 mmHg	Severe abdominal distension or pain that does not resolve spontaneously, or IAP > 20 mmHg
Nausea/vomiting	None, or asymptomatic with continuous gastrointestinal decompression	Nausea without vomiting	Nausea and vomiting (no gastrointestinal decompression required), or gastric residual volume (GRV) > 250 mL	Vomiting requiring gastrointestinal decompression, or GRV > 500 mL
Diarrhea	None	Loose stools 3–5 times/day with volume < 500 mL	Loose stools ≥ 5 times/day with volume 500–1,500 mL	Loose stools ≥ 5 times/day with volume ≥ 1,500 mL

GRV, Gastric Residual Volume; 1 mmHg = 0.133 kPa.

### 4.4 Novel objective monitoring techniques

#### 4.4.1 Gastrointestinal ultrasound

It provides a real-time, non-invasive method for assessing gastric motility by measuring the cross-sectional area (CSA) of the gastric antrum, calculating gastric emptying rates, and evaluating antral motility. Current evidence indicates that ultrasound assessment significantly surpasses traditional gastric residual volume aspiration in predicting feeding intolerance. Prospective studies have confirmed the high feasibility of using ultrasound for monitoring gastric residual volume (GRV) and feeding tolerance, establishing it as a superior alternative to aspiration methods (20). When combined with the acute gastrointestinal injury score, this technique effectively guides enteral nutrition therapy in critically ill patients (35). In particular, for neurocritically ill patients, the integrated use of bedside ultrasound antral motility index and enteral nutrition tolerance scores has demonstrated outstanding clinical utility (36).

#### 4.4.2 Gastric impedance monitoring

It is: a technique that continuously assesses gastric electrical activity and the process of gastric content emptying through the use of superficial electrodes. This method is advantageous due to its non-invasive nature and dynamic capabilities, making it particularly suitable for critically ill patients who are under deep sedation and unable to participate actively in examinations (37).

#### 4.4.3 Biomarkers

Serum levels of hormones such as motilin and cholecystokinin (CCK) are closely linked to gastric motility. A significant decrease in these hormone levels can serve as an early indicator of delayed gastric emptying and an increased risk of feeding intolerance (FI).

#### 4.4.4 Gastric emptying imaging

Specifically radionuclide labeling imaging, is considered the scientific “gold standard” for evaluating gastric emptying function. However, its application in intensive care unit (ICU) settings is severely constrained due to challenges such as radiation exposure, procedural complexity, and difficulties in equipment mobility (38).

## 5 Multidimensional management strategies for FI in sedated neurocritical patients

**FIGURE 2:**
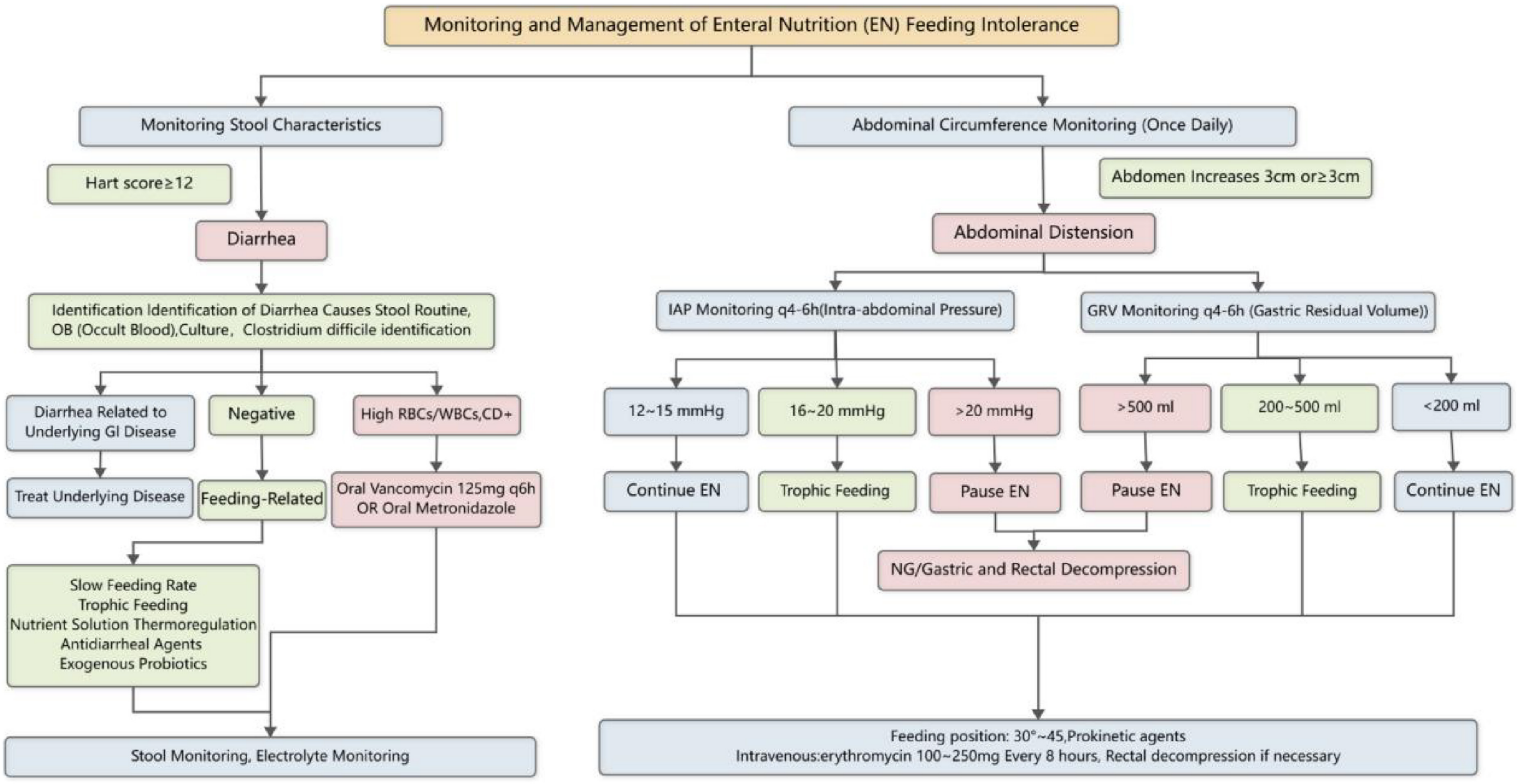
Algorithm for monitoring and management of enteral nutrition feeding intolerance in neurosurgical critically ill patients. OB, Occult Blood Test; IAP, Intra-abdominal Pressure; GRV, Gastric Residual Volume; EN, Enteral Nutrition; 1 mmHg = 0.133 kPa. Hart score (19).

Management should be tailored to the dual goals of maintaining cerebral protection and preserving gastrointestinal function (1, 39, 40) (Figure 2).

### 5.1 The initiation timing of enteral nutrition

Both the ASPEN/SCCM (2016) guidelines and the “Expert Consensus on Nutritional Support Therapy for Critically Ill Patients in China” advocate for the initiation of enteral nutrition within 24–48 h, asserting its superiority over parenteral nutrition (17, 41–43). Similarly, the European Society for Parenteral and Enteral Nutrition (ESPEN) (2019) recommends commencing enteral nutrition within 48 h for patients with traumatic brain injury (24). According to the nutritional guidelines of the European Society of Intensive Care Medicine (ESICM) (2017) (18), despite the lack of conclusive evidence from several recent randomized controlled trials regarding the definitive benefits of early enteral nutrition, expert opinion supports its early initiation in patients with traumatic brain injury, ischemic or hemorrhagic stroke, and spinal cord injury. However, enteral nutrition should be delayed in specific clinical scenarios, including uncontrolled shock, hypoxemia, severe acidosis, active gastrointestinal bleeding, gastric retention exceeding 500 mL over 6 h, intestinal ischemia, intestinal obstruction, and abdominal compartment syndrome. Additionally, enteral nutrition should be deferred for patients undergoing therapeutic hypothermia for severe conditions.

### 5.2 Selection of enteral nutrition formulations

#### 5.2.1 Selection criteria

For patients exhibiting normal gastrointestinal function, a whole protein formula is recommended as the preferred choice. In cases of gastrointestinal dysfunction, such as diarrhea or malabsorption, a short peptide or amino acid formula is advised, as these formulations either bypass the need for digestion or require minimal digestion for absorption (25, 44–48).

#### 5.2.2 Dietary fiber considerations

Soluble dietary fiber, such as guar gum or pectin, should be routinely incorporated into the enteral nutrition regimen, except in instances of intestinal obstruction or severe diarrhea (2). Multicenter studies have identified the early addition of dietary fiber (within 3 days of initiating enteral nutrition) as an independent protective factor against functional impairment (FI). Dietary fiber undergoes fermentation by colonic bacteria, producing short-chain fatty acids like butyric acid, which serve as the primary energy source for colonic mucosal cells. This process effectively maintains epithelial barrier integrity, modulates intestinal flora, and significantly reduces diarrhea incidence (49). The incorporation of dietary fiber represents a crucial, cost-effective, and efficient intervention in clinical practice.

#### 5.2.3 Diabetes-specific formula

For patients experiencing stress-induced hyperglycemia, a diabetes-specific formula characterized by low sugar content and high monounsaturated fatty acids may be selected to aid in blood glucose management (50).

#### 5.2.4 Other considerations

In neurosurgical critically ill patients, additional glutamine (GLN) supplementation is unnecessary when protein intake through enteral nutrition is adequate. However, for patients with severe traumatic brain injury, GLN supplementation may be considered if plasma GLN levels are reduced. In patients receiving standard total parenteral nutrition, attention should be given to GLN supplementation. It is not recommended to routinely incorporate EPA/DHA or employ high-dose enteral formulas rich in ω-3 fatty acids for intestinal nutrition in neurosurgical critically ill patients. Nevertheless, for parenteral nutrition, fat emulsions containing EPA+DHA (with a fish oil dose of 0.1–0.2 g⋅kg^–1^⋅d^–1^) may be considered (1).

### 5.3 Feeding route and infusion method

#### 5.3.1 Route selection

For patients unable to consume food orally and who present a heightened risk of intolerance or aspiration with gastric feeding (Table 4) (51), post-pyloric feeding methods, such as the insertion of a nasojejunal tube, are advised. Empirical evidence indicates that post-pyloric feeding substantially decreases the incidence of ventilator-associated pneumonia (1, 52).

**TABLE 4:** Aspiration risk assessment scale for critically Ill neurosurgical patients during enteral nutrition therapy.

Assessment item	1 Point	2 Points	3 Points
Age (years)	10–49	50–80	> 80 or < 10
Consciousness	Alert	Alert with sedation	Coma
Sputum	Scant	Abundant and thick	Abundant and thin
Comorbidities (Alzheimer’s disease, cerebrovascular accident, myasthenia gravis, Parkinson’s disease)	None	1 Condition	More than 1 condition
Diet	NPO (nil per os)	Regular diet	Liquid or semi-liquid diet
Body position	Semi-recumbent position ≥ 30°	Semi-recumbent position < 30°	Supine position
Water swallow test	Grade 1	Grade 2	Grade 3 or above
Artificial airway / mechanical ventilation	None	Present	/
Total score			

Risk stratification: 10–12 points = low risk; 13–18 points = moderate risk; 19–23 points = high risk.

#### 5.3.2 Infusion principles

It is highly advisable to employ a nutrition pump for continuous infusion. The initial infusion rate should be set at a slow pace (e.g., 20–30 mL/h), adhering to the principle of “progressing from slow to fast, and from small to large.” The dosage may be adjusted as necessary every 8–12 h, contingent upon the patient’s tolerance (2, 3, 53).

#### 5.3.3 Goal setting

To evaluate the impact of sedation and analgesia on energy requirements and gastrointestinal function in patients with severe neurological conditions during acute stress periods. The administration of sedation and analgesia has been observed to decrease energy demands and delay gastric emptying, irrespective of concurrent use of neuromuscular blocking agents. This can lead to an increased risk of feeding intolerance in deeply sedated patients. Opioid-based sedative and analgesic regimens may adversely affect nutritional metabolism by reducing gastrointestinal motility, causing gastric retention, constipation, gastroesophageal reflux, weight loss, and malnutrition, thereby potentially hindering neurosurgical treatment outcomes. Implementing a “low-calorie feeding” strategy, providing 60%–70% of the target energy intake, may mitigate risks of overfeeding and refeeding syndrome. A gradual transition to full feeding is recommended once the patient reaches a stable condition.

### 5.4 Pharmacological intervention

#### 5.4.1 Gastrointestinal motility enhancers

For patients experiencing delayed gastric emptying, pharmacological agents such as metoclopramide, mosapride, and erythromycin may be considered (2). Research indicates that administering a low dose of erythromycin can significantly ameliorate symptoms of gastroparesis (31). It is imperative to consider the potential side effects associated with these medications during their administration. For instance, erythromycin has been shown to prolong the QT interval, thereby elevating the risk of arrhythmias. Similarly, metoclopramide may precipitate extrapyramidal reactions, especially in patients who have experienced head trauma.

#### 5.4.2 Probiotics and prebiotics

In cases of diarrhea, after excluding infectious causes, the administration of probiotics to modulate gut flora or the use of synbiotics (a combination of probiotics and prebiotics) can aid in restoring intestinal microecological balance.

Sedative and analgesic drugs: The early cessation of medications that induce gastrointestinal paralysis, such as fentanyl and morphine, which impede intestinal peristalsis, is advantageous for enhancing gastric emptying and improving tolerance to enteral nutrition.

#### 5.4.3 Additional interventions

Correct hypoalbuminemia (via albumin supplementation and fluid restriction) to reduce intestinal mucosal edema, normalize electrolytes, and promote peristalsis—all of which alleviate abdominal distension (6, 19).

### 5.5 Implementation of non-pharmaceutical interventions

#### 5.5.1 Position management

It is recommended to maintain the elevation of the head of the bed between 30° and 45° during feeding and for 1 h post-feeding. This practice is recognized as the most straightforward and effective strategy for mitigating the risk of reflux and aspiration (54).

Regulation of Nutrient Solution Temperature, Concentration, and Administration Rate: The nutrient solution should be maintained at a temperature between 38 and 40°C using a heater to prevent gastrointestinal spasms induced by cold exposure (55). The concentration and administration rate of the nutrient solution should be adjusted in accordance with the patient’s gastrointestinal tolerance.

#### 5.5.2 Abdominal massage

For patients experiencing abdominal distension and constipation, gentle clockwise abdominal massage is recommended, as it can facilitate intestinal peristalsis and provide auxiliary benefits (36).

## 6 Conclusion

The management of feeding intolerance (FI) in sedated neurocritical patients is a discipline-specific challenge requiring integration of neurocritical care, pharmacology, and nutrition. Current evidence confirms high APACHE II score, elevated IAP, and hyperlactatemia as neurospecific independent FI risk factors, while early dietary fiber supplementation is protective. Clinical practice should prioritize early risk stratification, combine multimodal monitoring (IAP, GRV, tolerance scoring), and implement individualized intervention pathways to break the cycle between gastrointestinal failure and neurological deterioration.

Future research should emphasize precision and individualization by focusing on two key areas:

### 6.1 Development of predictive models

Utilizing machine learning technologies and integrating multi-omics data (such as the microbiome and metabolome) to create more accurate FI risk prediction models (56).

### 6.2 Optimization of sedation protocols

Investigating the Differential Effects of Various Sedation and Analgesia Regimens on Gastrointestinal Function, with a Focus on Comparing Dexmedetomidine and Opioids, and Identifying Drug Combinations that Achieve Effective Sedation with Minimal Gastrointestinal Impact.

### 6.3 Implementation of advanced monitoring technologies

Encourage the utilization of bedside ultrasound, electrical impedance tomography, and similar technologies to facilitate non-invasive, real-time monitoring of gastric emptying and intestinal motility. For instance, bedside ultrasound can evaluate gastrointestinal function impairment in critically ill patients and predict feeding tolerance, as demonstrated by the AGIUS examination score. Research indicates that increased echo density of the gastric antrum correlates with the severity of acute gastrointestinal injury (AGI). Patients exhibiting higher echo density in the gastric antrum following the initiation of enteral nutrition via a nasogastric tube are more prone to experiencing feeding intolerance (57–59).

High-quality clinical research is essential, and there is an urgent need for large-sample, multi-center randomized controlled trials (RCTs) focusing on neuro-intensive care patients. These studies are crucial to generate high-level evidence-based medical insights for specific intervention strategies, such as varying doses and types of dietary fibers and probiotic strains, and so on. By continuously enhancing our understanding of the underlying mechanisms and optimizing management strategies, we aim to ensure effective treatment of neurological disorders while maximizing the preservation of intestinal function, ultimately improving the overall prognosis and quality of life for neuro-intensive care patients.
